# An Unusual Clinical Presentation of Scrub Typhus

**DOI:** 10.7759/cureus.5568

**Published:** 2019-09-05

**Authors:** Vinoth Kumar Sethuraman, Kavitha Balasubramanian

**Affiliations:** 1 General Medicine, Indira Gandhi Medical College and Research Institute, Puducherry, IND

**Keywords:** scrub typhus, pneumonia, eschar, doxycycline

## Abstract

Scrub typhus is still underdiagnosed despite a resurgence in incidence as the clinical presentation is often atypical leading to low index of suspicion among clinicians. We report a case of a young girl presenting as lobar pneumonia and diagnosed as scrub typhus. Despite such a classical picture of community-acquired pneumonia on clinical presentation and radiological findings the patient was found to have scrub typhus serologically thereby posing a diagnostic dilemma. Upon serological confirmation, doxycycline therapy was initiated followed by a rapid and complete resolution of pneumonia, both clinically and radiographically. This case report highlights the importance of recognizing an uncommon clinical presentation of this common tropical disease and its prompt diagnosis and treatment.

## Introduction

Scrub typhus, also known as tsutsugamushi disease, is a bacterial infection transmitted by larval trombiculid mites from rodents often during cooler months. The causative organism is *Orientia tsutsugamushi*, an obligatory intracellular bacterium that leads to the formation of eschar at the inoculation site followed by fever, headache, myalgia, generalized lymphadenopathy, cough, gastrointestinal symptoms, transient hearing loss, and rash [1]. Further progression of the disease may manifest as acute respiratory distress syndrome, meningoencephalitis, gastrointestinal bleeding, acute renal failure, and coagulopathy [2]. Scrub typhus is still underdiagnosed in spite of increasing awareness due to the varied clinical manifestations of the disease and a high index of suspicion is required for the diagnosis. We present a case of lobar pneumonia due to scrub typhus which is an atypical presentation and the importance of its early management.

## Case presentation

**Figure 1:**
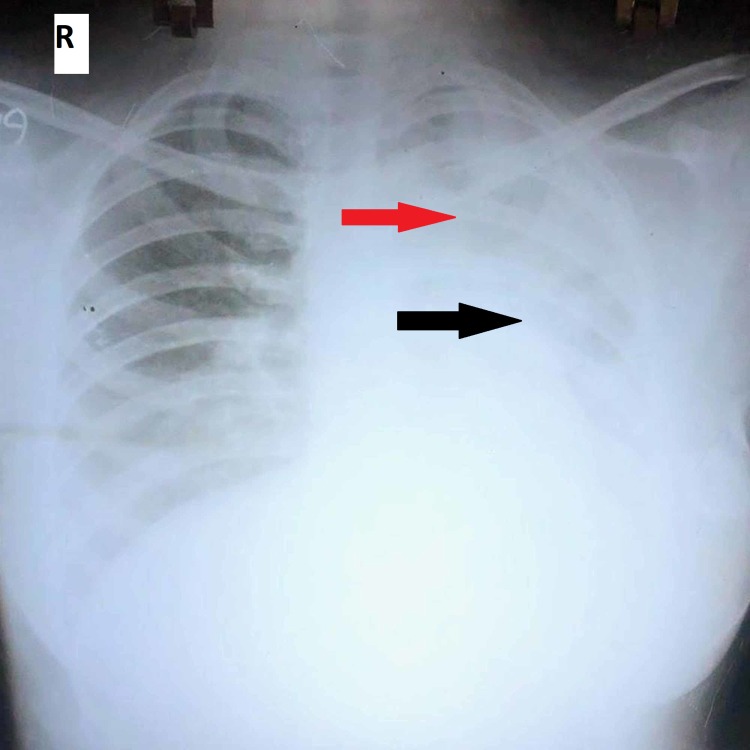
Chest radiograph on admission showing left upper (red arrow) and mid-zone (black arrow) heterogenous opacities with air bronchogram.

A 15-year-old girl from rural area was admitted with a history of fever with chills, headache, cough with scanty expectoration, and myalgia for five days duration. On examination, she was febrile (temperature 101°F), dehydrated and toxic appearing, tachypnoeic (26 breaths/min), and had pulse rate of 102/min and blood pressure of 100/60 mmHg with no skin rashes, eschar, and lymphadenopathy. Her systemic examination revealed tubular bronchial breathing in the left supraclavicular, infraclavicular, axillary, and suprascapular areas along with fine crepitations with SpO2 of 85%-90% in room air with normal cardiovascular status. Her abdominal and neurological examination was normal. The patient was diagnosed clinically to have left upper lobe consolidation with respiratory failure due to community-acquired pneumonia and started on injection ceftriaxone suspecting bacterial etiology along with oxygen after sending relevant investigations. Arterial blood gas analysis showed hypoxemia with respiratory alkalosis. Chest radiograph on admission revealed left upper and mid-zone heterogenous opacities with air bronchogram (Figure 1). Her investigations are summarized in Table 1.

**Table 1:** Investigations of the patient during her hospital stay. WBC, white blood cell; ESR, erythrocyte sedimentation rate; HBsAg, hepatitis B surface antigen; HIV, human immunodeficiency virus; AFB, acid fast bacilli; OD, optical density; CBNAAT, cartridge-based nucleic acid amplification test; MTB, mycobacterium tuberculosis; H1N1, hemagglutinin1 neuraminidase1.

	Day 1	Day 3	Day 5	Day 9	
WBC x 10^9^/L	7.4	7.3	8.2	8.5	
Neutrophils %	69	76	80	75	
Lymphocytes %	26	18	15	18	
Platelets x 10^9^/L	145	160	214	220	
ESR mm/hour	54	56	28	20	
Blood culture	Sterile	Sputum Gram stain	Negative	Sputum for AFB	Negative
Scrub IgM (Inbios International)	2.468 OD : Cutoff:<0.500	Sputum culture	No growth	Sputum for CBNAAT MTB	Not detected
HIV	Nonreactive	HBsAg	Negative	Throat swab for H1N1 Influenza Virus	Negative
Echocardiogram	Normal	Electrocardiogram	Sinus tachycardia	Ultrasound abdomen	Normal

**Figure 2:**
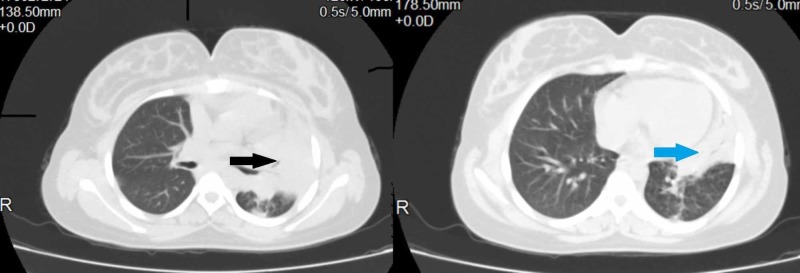
Computed tomography of the chest showing left upper lobe (black arrow) and lingular lobe (blue arrow) consolidation.

Contrary to our expectation, investigations revealed a normal white blood cell count with sputum negative for Gram stain and no growth in culture which is unusual in a case of bacterial pneumonia. Because of upper lobe involvement investigations for pulmonary tuberculosis were done which turned out to be negative. Even after two days of antibiotics the patient’s fever persisted and tachypnoea worsened and she became hypotensive (80/60 mmHg) and she was started on noninvasive ventilation and vasopressor. Computed tomography of the chest showed left upper and lingular lobe consolidation with air bronchogram with no pleural effusion (Figure 2).

**Figure 3:**
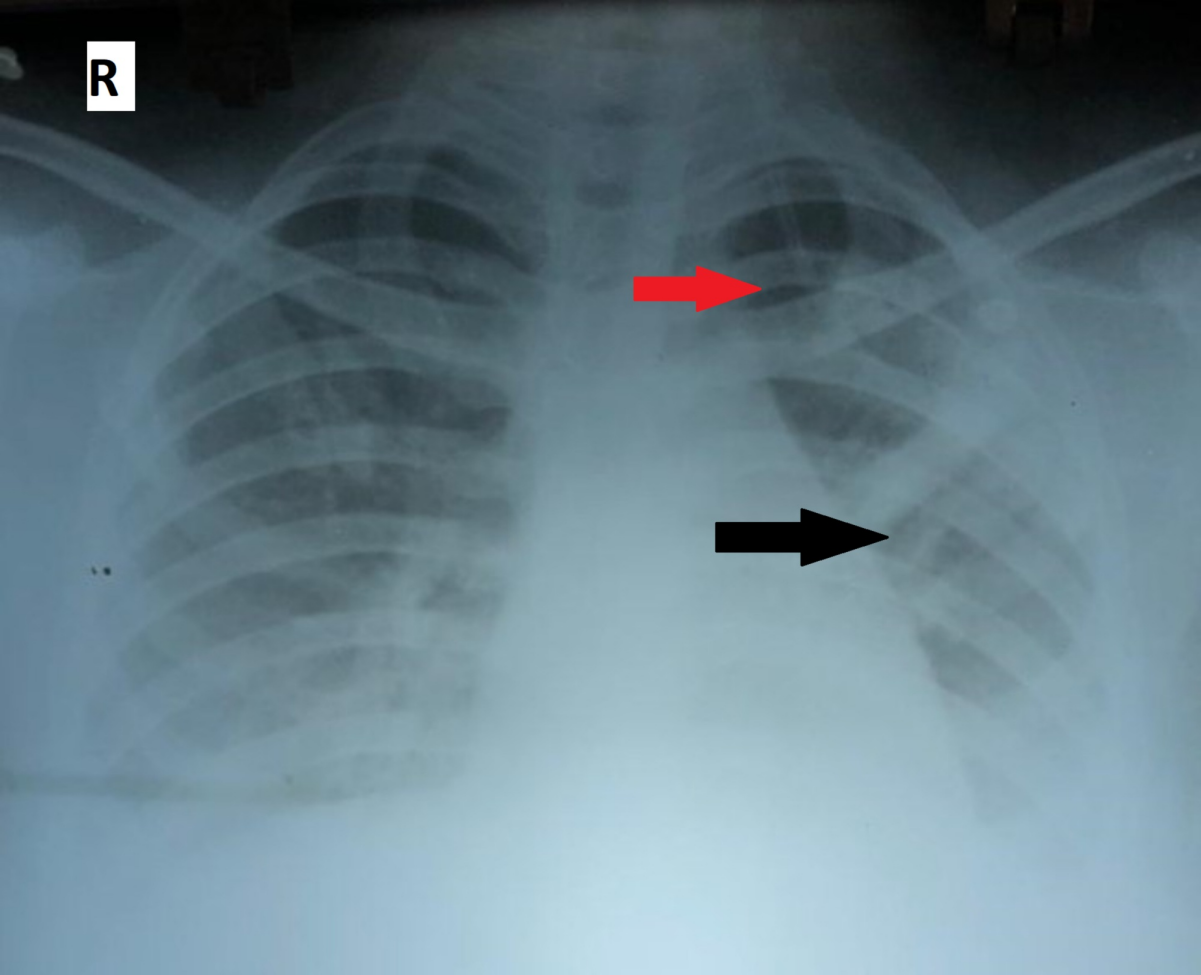
Chest radiograph before discharge showing complete resolution of left upper (red arrow) and mid-zone (black arrow) opacities.

As the patient is immunocompetent with normal white blood cell count and mild thrombocytopenia, our locality being a high prevalence area, scrub typhus was suspected and enzyme-linked immunosorbent assay (ELISA) for scrub typhus was done which came out to be positive. Oral doxycycline was added to the treatment regime and treated as scrub typhus pneumonia. Within 24 hours of adding doxycycline, fever spikes reduced and respiratory distress started improving. The patient was weaned of noninvasive ventilation on the seventh day and chest radiograph before discharge showed complete resolution of opacities which is unusual as radiological findings due to pneumonia lag behind the clinical improvement by few weeks (Figure 3). She was discharged home on the 10th day of admission on oral doxycycline for four more days and on follow-up visit after a week, she was asymptomatic.

## Discussion

Scrub typhus is one of the neglected and most common emerging and re-emerging diseases. Globally, over one billion people are at risk and an estimated one million cases of scrub typhus occur annually [3]. It is reported to be on the resurgence accounting for up to 50% of undifferentiated fever presenting to the hospital in some regions of India [4]. The mortality rate ranges from 30% to 45% if not diagnosed promptly and treated in endemic countries [5].

Scrub typhus presentation varies from mild and self-limiting undifferentiated fever to severe fatal condition. The patient commonly presents with acute flu-like symptoms, maculopapular rash, regional or generalized lymphadenopathy, and eschar. Eschar positivity is observed ranging from 7% to 97% among scrub typhus patients [6]. Eschar can be absent as in our patient or may be overlooked. In its severe form scrub typhus presents with an acute febrile illness with multi-organ dysfunction. Immunofluorescence assay (IFA) is the reference serological method for the diagnosis of rickettsial diseases and is considered a serological ‘gold standard.’ However, the cost and requirement of technical expertise limit its wide use. IgM ELISA is a commonly used test for screening and confirming the scrub typhus infection [7]. The antibiotics that are effective and recommended are doxycycline, chloramphenicol, and azithromycin [8].

Pulmonary involvement is a well-documented complication of scrub typhus infection. Respiratory complications of scrub typhus have been variably reported with interstitial pneumonia at one end of the spectrum to fatal acute respiratory distress syndrome (ARDS) at the other end [9-10]. Up to 58.4% of the patients may portray pulmonary involvement in the form of symptoms like cough and dyspnoea [11]. Basic pathology is interstitial pneumonia with or without vasculitis [12]. The incidence of chest radiographic abnormalities in patients with scrub typhus varies from 59% to 72% [13]. The most common radiological findings reported in the literature are interstitial pneumonia, bilateral diffuse areas of reticulonodular opacity, hilar adenopathy, pleural effusion, and focal atelectasis. Interstitial pneumonia has been found in most patients with pulmonary involvement. The presence of interstitial pneumonia is closely associated with morbidity and severity of disease for patients with scrub typhus [14]. Airspace consolidation is relatively uncommon and generally appears in the lower zone of both lungs. Our patient had a massive airspace consolidation involving the left upper and lingular lobes which is an unusual pulmonary manifestation of scrub typhus and has been reported once in literature [15]. Characteristic high-resolution computed tomography (HRCT) features of scrub typhus include ground-glass opacity predominantly in the lower zones, bronchial wall thickening, centrilobular nodules, and interlobular septal thickening [16]. Definitive treatment must be initiated without waiting for laboratory confirmation of scrub typhus based on clinical and epidemiological evidence as if left untreated it can be fatal and antibiotics may not be effective once complications set in.

## Conclusions

Lobar pneumonia which is commonly due to community-acquired pneumonia of bacterial etiology can also be a presenting feature of scrub typhus and clinicians should be aware of such a presentation. Conventional antibiotics for the treatment of pneumonia might not be beneficial for them and delay in the institution of appropriate antibiotic often leads to rapid deterioration in the clinical condition. In endemic regions, one should carefully look for eschar on physical examination as it is a highly specific clinical sign of scrub typhus that can guide the clinician and in its absence, serological testing should be done in atypical clinical scenarios as scrub typhus can present with a myriad of clinical manifestations.
